# Change in axial length after vitrectomy with silicone oil tamponade for rhegmatogenous retinal detachment

**DOI:** 10.1186/s12886-022-02433-8

**Published:** 2022-06-08

**Authors:** Jiemei Shi, Kaicheng Wu, Huiming Wen, Jiaojiao Wei, Yuan Zong, Jian Yu, Haohao Zhu, Chunhui Jiang

**Affiliations:** 1 Department of Ophthalmology and Vision Science, Eye and ENT Hospital, Fudan University, Shanghai, 200031 People’s Republic of China; 2 Department of Ophthalmology, People’s Hospital of Shanghai No. 5, Shanghai, 200240 People’s Republic of China

**Keywords:** Rhegmatogenous retinal detachment, Axial length, Vitrectomy, Silicone oil tamponade, Retrospective study

## Abstract

**Background:**

We aimed to explore the changes in the axial length and related factors after vitrectomy for rhegmatogenous retinal detachment (RRD).

**Methods:**

This study retrospectively evaluated patients who underwent vitrectomy with silicone oil (SO) tamponade for RRD and subsequent silicone oil removal at our clinic. Using a Zeiss IOLMaster 700, axial length was measured before vitrectomy for RRD and SO removal. The change in axial length (ΔAL) was calculated, and multivariate binary logistic regression analysis was performed to investigate the potential correlation between ΔAL and clinical factors, such as preoperative hypotony, extreme myopia, age, macular involvement, choroidal detachment, operation duration, and operation history.

**Results:**

In total, 213 eyes from 213 patients were included. The mean axial length changed significantly pre- and post-vitrectomy (25.98 ± 2.87 mm and 26.25 ± 3.07 mm, respectively, *P* < 0.001); the mean ΔAL was 0.37 ± 0.62 mm. Multivariate binary logistic regression analysis showed that preoperative hypotony and extreme myopia were significantly correlated with the ΔAL (*P* = 0.001 and *P* = 0.001, respectively). A higher proportion of hypotonic eyes had ΔAL ≥ 0.3 mm (33/76 in hypotony eyes and 32/137 in others; *P* = 0.003). A higher proportion of extremely myopic eyes also had a ΔAL ≥ 0.3 mm (23/46 in extremely myopic eyes and 42/167 in others; *P* = 0.002).

**Conclusion:**

For patients with RRD and cataracts, as axial length changed significantly after vitrectomy in those with hypotony or extreme myopia, secondary lOL implantation should be considered.

**Supplementary Information:**

The online version contains supplementary material available at 10.1186/s12886-022-02433-8.

## Background

Pars plana vitrectomy (PPV) has long been used to treat rhegmatogenous retinal detachment (RRD), [1] often combined with phacoemulsification [2]. However, there are still some controversies over whether an intraocular lens (IOL) should be implanted simultaneously [2, 3]. One procedure results in fewer surgery visits, faster visual recovery, and lower costs, [4, 5] although the disadvantage of postoperative refractive errors, especially myopic shift, is of concern [6, 7]. Several factors may contribute to postoperative refractive error, and underestimation of axial length (AL) may be the main source of this error [8]. Several studies have explored the change in AL after PPV for RRD; however, the results remain controversial [6, 9–11]. Some studies were limited by a small number of cases, [6, 9, 10] while some used ultrasound, which has been found to have low accuracy in RRD cases. The use of the IOLMaster 700 is reportedly more accurate than ultrasound for AL measurement in RRD eyes [12]. Hence, this study used an IOLMaster 700 to measure AL before and after PPV with silicone oil (SO) tamponade. The change in AL was assessed, and the potential clinical factors associated with larger AL changes were further analyzed.

## Methods

Patients who underwent PPV with SO tamponade and subsequent SO removal at our hospital between January 2016 and April 2021 were recruited for this study. Only patients whose retina was still attached 2 months after SO removal were included. This study was conducted according to the tenets of the Declaration of Helsinki. All patients provided written informed consent for participation in and publication of this study. All eyes underwent a standard 23/25-gauge PPV with SO (Oxane 5700, Bausch & Lomb Inc., Waterford, Ireland) injection. After a mean 5.32 ± 2.3 months, the SO was removed through the pars plana, 3 mm posterior to the limbus, the same location as it was injected. Patient demographics, medical history, and clinical characteristics were retrieved from their medical records. Any vitreoretinal procedure, such as scleral buckling and vitrectomy, performed before the PPV with SO injection for RRD was considered a vitreoretinal operation history. A thorough ocular examination was performed before each operation, including best-corrected visual acuity (BCVA) measurement, slit-lamp biomicroscopy, indirect ophthalmoscopic examination of the fundus, intraocular pressure (IOP) measurement with a non-contact tonometer (Nidek NT400, Nidek Co., Ltd., Aichi, Japan), and AL measurement using an IOLMaster 700 (version 3.01; Carl Zeiss Meditec, Jena, Germany). Change in AL (ΔAL) was calculated as follows: ΔAL =|postoperative AL – preoperative AL|. Eyes with an IOP less than 10 mmHg [13] or IOP difference between two eyes more than 4 mmHg (fellow eye – operated eye) were classified as hypotonic [14, 15]. Eyes with preoperative AL ≥ 28 mm were defined as extremely myopic [16]. Cases with duration for more than three months was defined as Chronic RD [17].

### Statistical analysis

Statistical analysis was performed using SPSS Statistics 20.0 for Windows (version 20.0, IBM Corp., Armonk, NY, USA). Continuous values are expressed as the mean ± standard deviation. Continuous variables were compared using the paired t-test and Student’s t-test. We compared clinical characteristics between those with or without ΔAL ≥ 0.3 mm using the chi-square test. Univariate binary logistic regression analysis was used for elucidating potential clinical factors that lead to ΔAL ≥ 0.3 mm and factors with a *P*-value of < 0.1 underwent multivariable logistic regression analysis. A *P*-value less than 0.01 was considered to be statistically significant.

## Results

There were 213 eyes of 213 patients (120 male and 93 female) with ages ranging from 13 to 79 years (mean 53.97 ± 13.10 years) that were included in this study. In 131 (61.5%) eyes, phacoemulsification was combined during PPV for RRD. Preoperatively, the mean IOP of the RRD eyes was significantly lower than that of the fellow eyes (12.67 ± 4.32 mmHg vs 14.92 ± 3.40 mmHg; *P* < 0.001), and the mean AL was 25.98 ± 2.87 mm (range: 21.60–34.83 mm). At the time of SO removal (mean 5.32 ± 2.3 months later), the mean IOP was 17.57 ± 5.46 mmHg, which was significantly higher than the preoperative IOP (*P* < 0.001). The postoperative mean AL was 26.25 ± 3.07 mm (range: 21.54–35.61 mm), which was also higher than the preoperative mean AL (*P* < 0.001) (Table 1).

**Table 1:** Demographic and clinical characteristics of patients

Variable		Patients (*n* = 213)
Age, mean ± SD		53.97 ± 13.10
Gender	Male	120
	Female	93
Macula involvement	On	79
	off	134
Duration between two operations(month)		5.32 ± 2.3
Vitreoretinal operation history	Yes	26
	No	187
AL^a^		
Preoperative AL (mm), mean ± SD		25.98 ± 2.87
Postoperative AL (mm), mean ± SD		26.25 ± 3.07
ΔAL (mm), mean ± SD		0.37 ± 0.62
IOP^b^		
Preoperative IOP (mmHg), mean ± SD	Operated Eyes	12.67 ± 4.32
	Contralateral Eyes	14.92 ± 3.40
Postoperative IOP (mmHg), mean ± SD	Operated Eyes	17.57 ± 5.46
	Contralateral Eyes	15.42 ± 3.43

^a^Statistical difference between pre-operated and post-operated AL (Paired T test), *P* values less than 0.001

^b^Statistical difference between operated and contralateral eyes both pre and postoperatively (Paired T test), *P* values less than 0.001

*IOP* intraocular pressure, *AL* axial length

The overall mean ΔAL was 0.37 ± 0.62 mm. Univariate binary logistic regression analysis revealed that preoperative hypotony (*P* = 0.003) and extreme myopia (*P* = 0.002) were related with a high incidence of ΔAL ≥ 0.3 mm. However, age (*P* = 0.222), macular involvement (*P* = 0.733), chronic RD (*P* = 0.144), choroidal detachment (*P* = 0.069), operation duration (*P* = 0.104), and operation history (*P* = 0.069) were not associated. Multivariate binary logistic regression analysis confirmed that preoperative hypotony and extreme myopia were two factors significantly correlated with a high incidence of ΔAL ≥ 0.3 mm (hypotony: *P* = 0.001; extreme myopia: *P* = 0.001) (Table 2).

**Table 2:** Binary logistic regression analyses of the association between Δ AL and clinical variables

	**Univariate analysis**			**Multivariate analysis**		
Variable	**OR**	**95% CI**	***P*-value**	**OR**	**95% CI**	***P*-value**
Age	0.986	0.965–1.008	0.222			
Macula involvement (off,1; on,0)	1.112	0.606–2.040	0.733			
Chronic RD (yes,1; no,0)	1.757	0.825–3.742	0.144			
Choroidal detachment (yes,1; no,0)	2.856	0.920–8.863	0.069			
Operate duration	1.105	0.980–1.246	0.104			
Vitreoretinal operation history (yes,1; no,0)	2.167	0.941–4.990	0.069			
Preoperative hypotony (yes,1; no,0)	2.518	1.379–4.597	0.003*	2.771	1.481–5.186	0.001*
Preoperative extreme myopia (AL ≥ 28 mm) (yes,1; no,0)	2.976	1.515–5.848	0.002*	3.306	1.638–6.672	0.001*

*OR* odds ratio, *CI* confidence interval, *AL* axial length, *RD* retinal detachment

^*^P values less than 0.01

Eyes with preoperative hypotony had a higher rate of ΔAL ≥ 0.3 mm (33/76, 43.4%) than those without (32/137, 23.4%) (*P* = 0.003). Eyes with extreme myopia had a higher rate of ΔAL ≥ 0.3 mm (23/46, 50%) than those without (42/167, 25.1%) (*P* = 0.002). The risk of ΔAL ≥ 0.3 mm among subjects with hypotony was, on average, 2.771-fold higher than that among subjects without hypotony (odds ratio [OR] = 2.771, 95% confidence interval [CI] 1.481–5.186). The ORs of ΔAL ≥ 0.3 mm among patients with extreme myopia were, on average, 3.306-fold higher than those among patients without extreme myopia (OR = 3.306, 95% CI 1.638–6.672) (Table 2).

In 15 eyes that had both preoperative hypotony and extreme myopia, the ΔAL was 0.46 ± 0.37 mm (preoperative AL: 30.73 ± 1.92 mm vs postoperative AL: 31.12 ± 1.88 mm; *P* = 0.005), while the ΔAL in eyes with neither hypotony nor extreme myopia was 0.20 ± 0.29 mm (preoperative AL: 24.76 ± 1.45 mm vs postoperative AL: 24.83 ± 1.53 mm; *P* = 0.034). Eyes with both hypotony and extreme myopia had higher rates of ΔAL ≥ 0.3 mm (8/15 in eyes with both and 17/106 in the eyes with neither; *P* = 0.003) (Table 3).

**Table 3:** ΔAL in eyes with preoperative hypotony or not, in eyes with extreme myopia or not

		n	ΔAL ≥ 0.3 mm
Preoperative IOP	hypotony	76	33/76
	Not hypotony	137	32/137
*P* value^a^	0.003*		
Preoperative AL(mm)	extreme myopia	46	23/46
	Not extreme myopia	167	42/167
*P* value^a^	0.002*		
extreme myopia & hypotony	Both	15	8/15
	Neither	106	17/106
*P* value^a^	0.003*		

*AL* axial length, *IOP* intraocular pressure

* P values less than 0.01, ^a ^Statistical difference of eyes with hypotony or not, with extreme myopia or not (chi-square test)

## Discussion

A total of 213 eyes of 213 RRD patients were included in this study. The mean ΔAL after PPV was 0.37 ± 0.62 mm, and the ΔAL was closely and positively correlated with preoperative hypotony and extreme myopia. Theoretically, a positive change in AL would lead to a myopic shift and a negative shift to a hypermetropic one. In 107 hypotonic or extremely myopic eyes, AL had an average increase of 0.46 mm, approximately 1.15 D myopia shift. So for cases with hypotony or extreme myopia, this 1 D should be considered in the calculation of IOL.

PPV combined with phacoemulsification has been widely used in the treatment of RRD, but the refractive results are not always optimal [6, 7]. Accurate AL measurements are essential for the IOL power calculation. Both A-scan ultrasonography and assessment using the IOLMaster have been used in clinical practice. The IOLMaster measures the distance from the front of the cornea to the retinal pigment epithelium, [18] while A-scan ultrasonography measures the distance from the cornea to the internal limiting membrane, which has led to AL underestimation in previous studies [3]. In addition, changes in the refractive medium can also lead to inaccurate A-scan ultrasonography measurements [19]. The IOLMaster uses a technique based on partial coherence interferometry and is considered to be more accurate [20]. The new non-contact, swept-source-based IOLMaster 700, which provides a deeper scan depth and faster scan speed, [21] shows great repeatability and reliability for AL measurement, [22] and thus was used to study ΔAL in this study.

Previous studies have found ΔAL to be between 0.1 and 0.63 mm postoperatively; [9–11, 23] a ΔAL of 0.37 mm was reported in the current study, which is within this range. Analysis found that this change in AL was significant. Previously, Mukhtar et al. [10] and Liu et al. [9] reported a significant increase in AL after PPV for RRD, while Huang et al. [11] and Kang et al. [6] found no significant ΔAL. After close inspection of the data, it was noticed that Huang et al. [11] in fact reported a ΔAL of 0.63 mm postoperatively (preoperative AL 24.15 mm and 6-month postoperative AL 24.78 mm); however, they defined a P-value less than 0.005 as statistically significant. Kang et al. [6] only recruited macular-on RRD patients with BCVA ≥ 0.7, while patients with longer AL (AL ≥ 28 mm) were not recruited. However, myopia has a high prevalence in Asian populations, [24] and a large study in Taiwan showed that 10.51% of RRD patients were highly myopic (> − 6.0 D) [25]. In contrast, nearly half of the RRD patients were macular-off [26]. In the current study, 46 eyes were extremely myopic, one-third (76/213) were hypotonic, and half of the eyes (134/213) were macular-off, which may explain the variation among studies. When considering the 106 eyes that had neither hypotony nor extreme myopia preoperatively, the change of AL was also non-significant (pre: 24.76 ± 1.45 mm; post: 24.83 ± 1.53 mm; *P* = 0.034), which is in line with the results of Kang et al. [6].

It has been reported that a difference of 0.30 mm is correlated to a clinically significant 0.75-D error in the IOL power calculation, [27] and thus, 0.30 mm was used as the standard to separate the ΔAL in our study. Multivariate logistic regression analysis revealed that hypotony and extreme myopia were closely and positively correlated with increased ΔAL (Table 2). Zhang et al. [28] and Cho et al. [29] also reported that the change in IOP was related to increases in AL. In their studies, a 1.7–2-mmHg increases in postoperative IOP resulted in a 0.36–0.43-D myopic shift [28, 29]. Similarly, in our study, a mean increase of 5 mmHg of IOP was correlated with a 0.27-mm increase in AL (approximately 0.65-D myopic shift) [27, 29]. RRD is often accompanied by a reduction in IOP [30]. It was reported that eyes with uncomplicated unilateral retinal detachments have a mean IOP 1.3–3.5 mmHg lower than the fellow eyes, [31–34] and our study showed that 207 unilateral RRD eyes have a mean IOP 2.34 mmHg lower than the fellow eyes (*P* < 0.001) (Supplementary Fig. 1). Therefore, in hypotonic eyes, AL may be more likely to be underestimated preoperatively.

Another important factor associated with ΔAL was extreme myopia. Previously, Jee et al. [35] found that the ΔAL was significant in highly myopic eyes (0.46 ± 0.28 mm; *P* = 0.043) and non-significant in non-high myopic eyes (0.11 ± 0.34 mm; *P* = 0.135). Jeoung et al. [8] reported that in eyes with an AL of > 26 mm preoperatively, postoperative AL increased significantly (0.25 ± 0.23 mm; *P* < 0.05). Several factors may contribute to the change in AL in highly myopic eyes, such as the thickness of the sclera. Globe wall stress [36] can be calculated by IOP*(r/2t)**,** in which “r” is AL/2, and “t” is the wall thickness**,** which shows that stress is positively related with AL, and negatively with eye wall thickness. As a result, the same IOP change may lead to more stress changes in highly myopic eyes, which have a higher AL and thinner eye wall; this may explain the increased changes in AL.

Patients with RRD and cataracts can be treated with combined PPV and phacoemulsification, and the IOL may be implanted during the same procedure, or later. Our results suggest that AL would change for patients with hypotony or extreme myopia. And for these patients, it is better to re-measure the AL after the retina is reattached. This will result in a more accurate IOL calculation. The present study was limited by its single-center design, limited number of patients, and inclusion of only Chinese patients who received SO tamponade. Thus, the results require further verification.

## Conclusions

In the present study, we analyzed AL measured by the IOLMaster 700 in eyes with RRD before vitrectomy and SO removal. AL changed after vitrectomy for RRD, especially in cases with hypotony or extreme myopia, which suggests that secondary IOL implantation should be considered in these cases if phacoemulsification was considered necessary during the treatment of RRD.

## Supplementary Information

**Additional file 1:** **Supplement Fig. 1.** Intraocular pressure (IOP) changes following vitrectomy with silicon oil (SO) tamponade  in 207 unilateral RRD eyes. There is a significant difference between pre and post operation IOP in RRD eyes (*P*<0.001). And RRD eyes have a lower IOP than the fellow eyes preoperation (*P*<0.001). Paired t-test was used. *RRD* rhegmatogenous retinal detachment 

## Data Availability

All data relevant to the study are available from the corresponding author on reasonable request.

## Abbreviations

ΔAL: Change in axial length
AL: Axial length
BCVA: Best-corrected visual acuity
CI: Confidence interval
IOL: Intraocular lens
IOP: Intraocular pressure
OR: Odds ratio
PPV: Pars plana vitrectomy
RRD: Rhegmatogenous retinal detachment
SO: Silicone oil
