# Supplementary figures and images for: Change in axial length after vitrectomy with silicone oil tamponade for rhegmatogenous retinal detachment
Source: BMC Ophthalmol. 2022 Jun 8;22:257. doi: 10.1186/s12886-022-02433-8 (PMC9175333; doi:10.1186/s12886-022-02433-8)

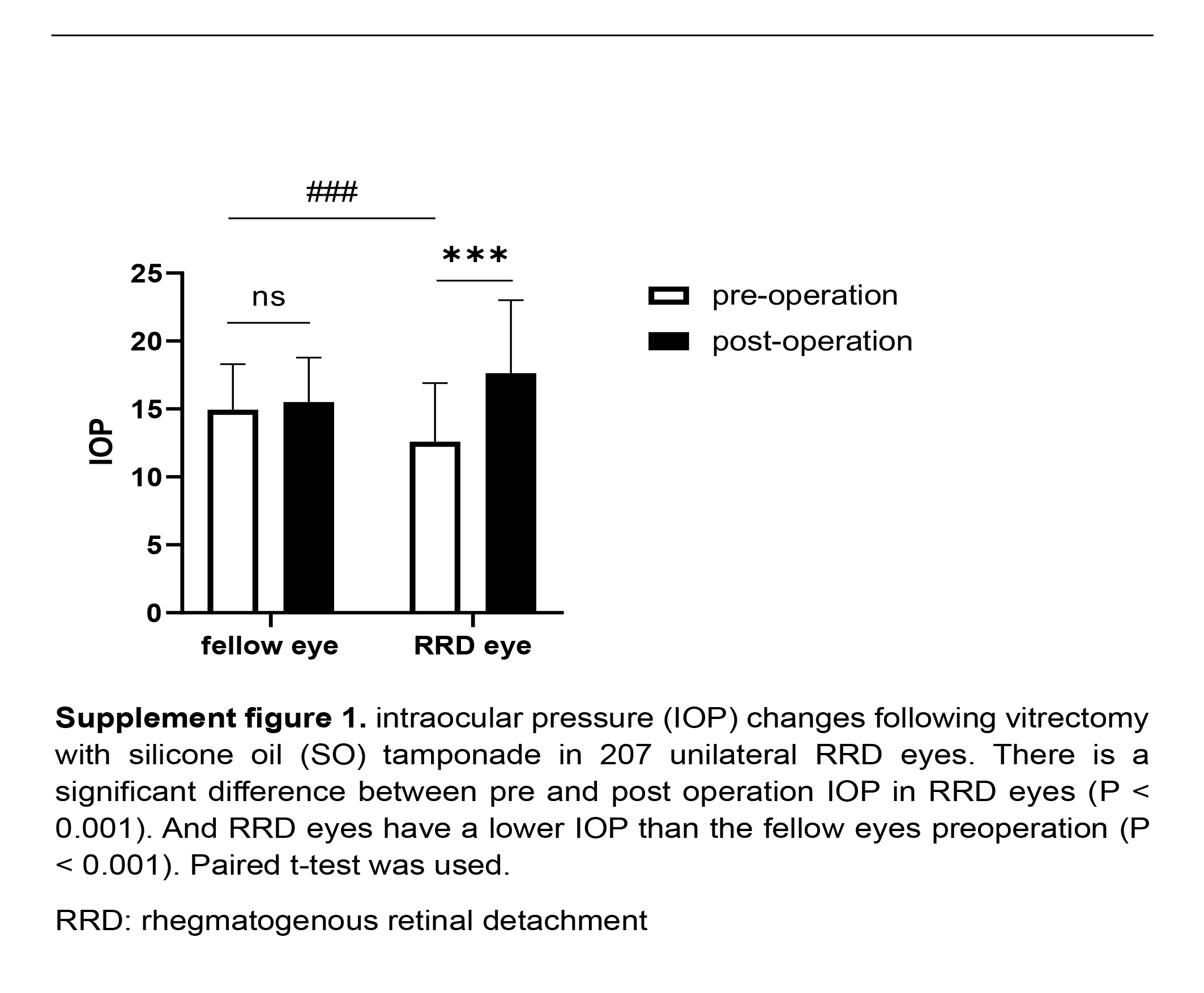

Supplement: Supplementary file 1 — Additional file 1: Supplement Fig. 1. Intraocular pressure (IOP) changes following vitrectomy with silicon oil (SO) tamponade in 207 unilateral RRD eyes. There is a significant difference between pre and post operation IOP in RRD eyes (P<0.001). And RRD eyes have a lower IOP than the fellow eyes preoperation (P<0.001). Paired t-test was used. RRD rhegmatogenous retinal detachment [file 12886_2022_2433_MOESM1_ESM.tif]
